# Efficacy of the Web-Based Gamified Infection Control Training System on Practices for Health Care Workers in Residential Care Homes: Clustered Randomized Controlled Trial

**DOI:** 10.2196/71593

**Published:** 2025-11-27

**Authors:** Angela Y M Leung, Doris Y P Leung, Terence K Lau, Justina Y W Liu, Teris Cheung, Daphne S K Cheung, Simon C Lam, Eliza M L Wong, Mimi M Y Tse, Alex Molassiotis

**Affiliations:** 1 School of Nursing Hong Kong Polytechnic University Hong Kong China (Hong Kong); 2 WHO Collaborating Centre for Community Health Services School of Nursing Hong Kong Polytechnic University Hong Kong China (Hong Kong); 3 Research Institute for Smart Ageing Hong Kong Polytechnic University Hong Kong China (Hong Kong); 4 Research Centre for Assistive Technology Hong Kong Polytechnic University Hong Kong China (Hong Kong); 5 School of Nursing and Midwifery Faculty of Health Deakin University Melbourne Australia; 6 Centre for Quality and Patient Safety Research/Alfred Health Partnership Institute for Health Transformation Deakin University Melbourne Australia; 7 Tung Wah College Hong Kong China (Hong Kong); 8 Hong Kong Metropolitan University Hong Kong China (Hong Kong); 9 College Arts, Humanities and Education University of Derby Derby United Kingdom

**Keywords:** clustered randomized controlled trial, gamification, hand washing, infection control training, personal protective equipment, residential care homes

## Abstract

**Background:**

Staff working in residential care homes (RCHs) have played a significant role in preventing the spread of infection among residents, visitors, and staff. Providing continuous professional training to the staff is essential. Current infection control training mostly rests on short educational talks or one-to-one reminders in the RCHs. A blended mode of online interactive games and face-to-face consultations was now proposed as a new way to conduct infection control training in the RCHs.

**Objective:**

This study aims to assess the efficacy of the Blended Gaming COVID-19 Training System (BGCTS) on infection control practices and self-reported knowledge, attitude, and practices of standard precautions among health care workers in RCHs.

**Methods:**

A 2-arm, single-blinded, parallel cluster randomized controlled trial was designed, and 30 RCHs were recruited and randomized into an intervention group to receive the BGCTS and a control group to receive usual care on infection control training. Due to the COVID-19 pandemic and infected cases in the homes, 17 RCHs refused or delayed the on-site observations. The BGCTS intervention, developed based on “The COVID-19 Risk Communication Package for Healthcare Facilities” of the World Health Organization, consists of two parts: (1) an eHealth mode of a 120-minute web-based training system covering 8 topics, delivered in short-clip videos and games, and (2) two 30-minute face-to-face interactive sessions for concept clarification. The 2 infection control practices, “use of gloves and personal protective equipment (PPE) and performing respiratory hygiene” and “hand rub,” were assessed by on-site unobtrusive observations, and self-reported infection control practices and knowledge and attitude toward infection control were measured via online survey post intervention.

**Results:**

A total of 212 staff from 13 RCHs were involved in the analysis, with 7 RCHs from the intervention group (n=114) and 6 RCHs from the control group (n=98). A significantly greater increase in the proportions of proper use of gloves and PPE and respiratory hygiene performance (β=.195, 95% CI 0.046-0.344; *P*=.02) and properly performed hand rub (β=.068, 95% CI 0.005-0.132; *P*=.04) was observed in the intervention group. The changes in the self-reported outcomes were not statistically significant.

**Conclusions:**

BGCTS improved RCH staff’s performance in 2 infection control practices by objective measurement, “gloves and PPE use and performance in respiratory hygiene” and “hand rub.” BGCTS was shown to be an effective training, although it was a 2-week intervention. The BGCTS did not perform better than infection control briefing sessions in self-reported infection control knowledge, attitude, and practices. This electronic-based infection control training with 2 intensive interactive sessions has good potential to be adopted as regular training in RCHs.

**Trial Registration:**

Clinicaltrials.gov NCT04783025; http://clinicaltrials.gov/ct2/show/NCT04783025

## Introduction

Residents in residential care homes (RCHs) are at a high risk of being infected by severe acute respiratory diseases (SARD) due to their advanced age, associated comorbidities, and state of dependence [1]. The price paid for an outbreak in RCHs is high. The pace at which SARD is spreading in RCHs is alarming. In a case study in Washington, after a first resident in RCHs was diagnosed with SARD, 101 residents, 50 health care personnel, and 16 visitors were infected, and the cases were proven to be epidemiologically linked [2]. One outbreak of a cluster of SARD in one RCH was enough to fill all of the beds set aside for SARD cases in a whole province in Korea [3]. Based on such evidence and many other reports around the globe, it appears clear that there is an urgent need to develop and implement an effective strategy to prevent the spread of infectious diseases in RCHs [4].

Staff working in RCHs have played a significant role in responding to the outbreak, particularly in preventing the spread of infection among residents, visitors, and staff. RCH staff have the responsibility to protect the residents, themselves, and visitors. Training the staff in RCHs is crucial because they are the gatekeepers of the safety of the residents. Unfortunately, most RCHs are unable to educate and train their employees due to a lack of resources [3]. It is suggested that governments provide an educational system for health care personnel in RCHs [3].

Globally, there is a lack of infection control training for staff in RCHs; even if training is provided, the existing online training programs are generic in nature, not specifically for SARD or COVID-19. Australia makes available e-learning modules to provide information on the principles of infection prevention and control in Australian health care settings [5]. These e-learning modules are suitable for all health care workers, including staff of RCHs. They include information on the principles of infection prevention and control; risk management systems for infectious agents and infectious diseases; basic microbiology and multiresistant organisms; cleaning, disinfection, and sterilization; infectious agent health screening and the immunization of health care workers; the investigation and management of outbreaks; the management of occupational exposure; risk management in renovation, repairs, and redevelopment; basic epidemiology; and surveillance and quality improvement. However, none of the material is specific to SARD or COVID-19. Only links to online resources about COVID-19 are provided, but no training is provided to RCH staff. A similar situation was noted in the United Kingdom and Hong Kong.

Infection control is a core element in preventing the spread of respiratory infections. However, low compliance with infection control practices poses significant challenges in the long-term care setting; in particular, nonmedical professionals were less frequently adherent to these practices than medical professionals [2,6,7]. Before the outbreak of the COVID-19 pandemic, various educational interventions for infection control had been developed. A recent systematic review (SR), based on a narrative synthesis of 11 interventional studies, suggested the preliminary benefits of educational interventions on attitudes and practices related to respiratory infection prevention. Meta-analytic results on 3 studies further showed significant improvements in knowledge about preventing respiratory diseases, although the evidence was of low certainty. The review also suggested that the use of multiple resources that can favor learning and stimulate hearing, vision, and touch in educational interventions may produce better results [8]. Despite these favorable findings, none of the 11 studies included RCH staff, which limits the generalizability to the long-term care facilities setting. Another SR reviewed articles evaluating the effect of infection prevention and control measures in the long-term care facilities setting and concluded that hand hygiene and oral hygiene have a beneficial effect on infection rates in the nonoutbreak setting. In contrast, infection prevention and control bundles seemed to be promising in an outbreak setting, but the finding was based on nonexperimental studies with a high risk of bias [9]. More recently, studies have been conducted to evaluate the impact of educational interventions that were developed in response to the COVID-19 pandemic. For example, an educational training program targeting health care workers in isolation centers could increase knowledge, attitude, and practices of infection prevention and control for COVID-19 [10]. Another study tested the effect of hand hygiene training among health care workers in a respiratory disease hospital and reported significant improvements in compliance with hand hygiene protocol and knowledge [11]. Other studies tested video-based educational interventions on knowledge, attitude, and practices during the COVID-19 pandemic, with promising results [12,13]. Again, these studies usually had used a pre-post study design without a comparison control group, affecting the internal validity of the study findings. The literature indicates that more studies with high quality are in urgent need for testing educational interventions in infection control, in particular to respond to outbreaks of SARD in the future.

Gamification is widely used as an educational tool in different disciplines. Various reviews have highlighted the wide applications and benefits of adopting gamification as a multidisciplinary educational tool. Gamified interventions have been applied across multiple domains, including science, technology, engineering, mathematics, linguistics, business, and medicine, to improve participants’ learning motivation [14]. A recent pre-post interventional study has applied gamified infection control training to health care workers in the hospital setting and reported significantly increased hand-rub consumption in a gastroenterological ward [15]. Furthermore, there are several potential benefits of the use of gamified training in infection control, in particular in the COVID-19 context. First, gamified training for infection control helps learners to understand the relationship between the various steps in infection control. If a learner does not strictly follow the infection control practices, widespread infection will occur in an RCH. If such a situation had taken place in reality, the residents would have suffered real harm. Second, gamified training provides feedback to learners, which helps them to understand the serious consequences of improper infection control practices and provides opportunities for improvement. Third, learners can choose to review the contents in a playful way, resulting in an increase in engagement in training. In summary, the gamified training for COVID-19 infection control provides a safe and flexible learning environment for the staff of RCHs to learn and practice decision-making.

In Hong Kong, infection control training is available largely to health care personnel working in hospitals or clinics and other health care settings. The Hong Kong Training Portal on Infection Control and Infectious Diseases offers programs only to staff from hospitals or clinics and other health care settings [16]. Since all RCHs in Hong Kong are under the jurisdiction of the Social Welfare Department or are privately operated, health care personnel working in RCHs are not eligible to receive this kind of infection control training. Overall, 2 universities are offering year-long infection control certificate programs to health professionals; the contents focus mostly on the daily management of infection control measures in hospitals and clinics [17]. In addition, the high turnover rate and demanding working time commitment in RCHs [18] also call for a flexible, interesting learning module in delivering infection control educational programs. Reviewing the existing infection control training offered around the globe, it is clear that there is a significant gap in the area of providing training in the prevention and control of SARD infections for health care personnel working in RCHs.

It was reported that nearly 5 billion people in the world have a mobile device [19]. Most staff in RCHs own a smartphone and have the technological competence to manage digital information and an electronic device [20]. Hence, smartphones and electronic devices are good platforms for training. In view of the demand for infection control training for staff in RCHs, the widespread use of smartphones, and the significant gaps in knowledge about the efficacy of infection control training on their actual practices, we developed a system called “Blended Gaming COVID-19 Training System (BGCTS)” for the staff of RCHs. Both electronic learning (with games and interactive quizzes) and face-to-face counseling sessions were used to help RCH staff to improve their infection control practices.

This study aims to assess the effect of the BGCTS on infection control practices among all staff in RCHs. The specific objectives of the study include:

1. To assess whether more health care workers in the intervention group performed proper infection control practices (objectively measured in unobtrusive on-site observations) than those in the control group after receiving infection control training.

2. To assess whether there were greater improvements in self-reported infection control knowledge, positive attitudes toward infection control, and infection control practices in health care workers in the intervention group than those in the control group.

## Methods

### Design and Setting

This is a 2-arm, single-blinded, cluster randomized controlled trial (RCT) conducted at RCHs in Hong Kong. The clusters of this study were RCHs in Hong Kong. Health care providers working in the same RCH formed a cluster to avoid potential contamination across study arms due to the sharing of the intervention content among staff in the same RCH. A total of 2 assessments were conducted at baseline and post intervention, respectively. This study adhered to the reporting guidelines of the Consolidated Standards of Reporting Trials (CONSORT) with an extension to cluster RCTs (Multimedia Appendix 1) [21].

### Participants

Inclusion criteria for participants were adult health care personnel who (1) provided direct care to the residents in the RCH, (2) were able to read Chinese, (3) possessed electronic devices (smartphones or tablets) for downloading the BGCTS, and (4) knew how to and were able to swipe the screens of electronic devices with their fingers. Exclusion criteria included (1) temporary staff who were going to cease their employment, be on maternity leave, or on a long vacation during the data collection period; (2) students or trainees (including nursing students, physiotherapy students, and health-related trainees), because training institutions were responsible for their training, and such training might affect their performance; (3) volunteer personnel who came to the RCHs at a specific period (eg, to organize activities for the residents during festivals); (4) clerical and administrative staff, kitchen staff, security staff, and engineering and facilities management staff; and (5) ambulance officers who came to the RCHs to take residents to or from hospitals.

The sample size was determined based on infection control practices. An observational study conducted by the project team in 2019 showed that the mean rate of compliance with hand hygiene measures among health care workers in RCHs was about 10% [22]. Assuming that the hand hygiene compliance rate in the control group would increase to 20% (ie, an increment of 10%) because of the COVID-19 pandemic and that the BGCTS could boost the compliance rate of the intervention group to 40% (ie, an increment of 30%), 138 health care workers would be needed to achieve 80% power at a 5% confidence level in an RCT. For a cluster RCT, assuming that 10 health care providers could be recruited from each RCH, that the intrainstitutional correlation (as measured by the intraclass correlation coefficient) was 0.04, and hence the design factor = 1+(10–1) × 0.04 = 1.36, the total sample size required was 188 (=138×1.36) participants or 20 RCHs. Further, allowing an attrition rate of 30% post intervention, we planned to recruit 30 RCHs to achieve the target sample size.

### Randomization and Masking

The clusters were randomly allocated in a 1:1 ratio to one of the two arms: (1) web-based gamified infection control training for 2 weeks and (2) usual care of a 30-minute infection control briefing. A statistician, who was not involved in the participant recruitment, generated a list of random numbers for group assignment using an online randomizer [23] and concealed the sequence using opaque sealed envelopes, which were opened by the research assistant during the group allocation procedure. The outcome assessor was blinded to the group assignment to avoid undue influence during the data collection process.

### Study Arms

#### The Intervention—BGCTS

The health care providers in the intervention group received a web-based gamified infection control training program (BGCTS) on an individual basis. The aims of the BGCTS are to promote the principles of infection control and reinforce the actions needed to stop the spread of COVID-19 within RCHs and how to provide care for those residents suspected of having COVID-19. The BGCTS has eight topics, namely (1) preparing for COVID-19 at your health care facility, (2) managing patients with suspected or confirmed COVID-19 at your health care facilities, (3) protecting yourself at work from COVID-19, (4) personal protective equipment (PPE) according to health care activities, (5) communicating with patients with suspected or confirmed COVID-19, (6) information about COVID-19, (7) coping with stress, and (8) my 5 moments for hand hygiene. These contents were developed based on the evidence-based contents from the World Health Organization (WHO) guideline “The COVID-19 Risk Communication Package for Healthcare Facilities” [24] and the infection control guidelines for care facilities developed by Hong Kong Government [16,25], with modifications based on feedback from stakeholders (Multimedia Appendix 2).

Elements of game design (ie, short video clips and interactive video games in the form of quizzes) in nongame environments (RCHs) within nongame contexts (infection control training) were adopted in the program development. In one of the games, the participants were asked to choose the essential PPE for their work at different sites in the RCHs. For example, in the triage scenario, where the staff received relatives or visitors who intended to enter the RCHs, staff should put on a medical mask only. A score as a reward was given for giving the correct answer to the question. Scores and a progress bar were shown to the staff so as to motivate their continuous participation in the training.

The participants were asked to complete the 8 topics in 2 weeks via the web-based system, which can be operated on laptops or mobile phones. They were encouraged to learn each topic independently in 15 minutes, resulting in a total of 120 minutes to complete all 8 topics in 2 weeks. In addition, in each week, a 30-minute face-to-face or Zoom (Zoom Communications, Inc) interactive session after they played the games was provided to allow the participants to clarify the infection control concepts with the infection control officer, the nurse who was trained for infection control, of the RCH. The final score achieved by the participant was shown on the screen and was also recorded in the scoring system so that the infection control officer can check which questions the participant could not answer correctly. Each participant was given an individual account with a unique username and password to access the platform [26]. The password could be reset by clicking the button “forgot the password.” Then, a new password would be sent to the participant’s email. Access to the platform was unlimited in the designated 2-week period. If the participant stopped playing the game at a particular point, the system had the record. When the participant played the game again, the system would continue the game and let the participant play the game at the point where the game was stopped. Online reminder was not made; however, the nurse managers in the RCHs verbally reminded the participant to complete the training within the designated period. No subscription fee to the BGCTS was charged in this study; however, a nominal subscription fee will be applied in the future to maintain the operation of the system.

#### Usual Care

Participants in the control group received the infection control briefing session by the infection control officer, who was either a registered nurse or a health worker appointed to handle matters related to infection control in the RCH. The appointment of an infection control officer is a licensing requirement of RCH in Hong Kong [27]. However, the briefing session was usually irregular, unstructured, and determined by infection control officers, depending on when the infection control officers receive the information about infection control practice from the Centre for Health Protection of the Hong Kong Government [25]. In addition, the format of the briefing session was mostly face-to-face in the form of a verbal announcement or short talk that lasted for less than 30 minutes, determined by the infection control officer. It is also common that the infection control information was delivered in the form of posters or a written document and circulated to all staff in the RCH, partly due to the high turnover rate in RCHs [18].

### Measures

#### Overview

The study outcomes were measured at 2 time points, baseline and post intervention, following group allocation. The primary outcome of the study was infection control practices performed by RCH staff, which is an objective measure made by on-site unobtrusive observations by trained research nurses (offline). Secondary outcomes included self-reported infection control practice and knowledge and attitudes toward infection control [28], which were included in an online self-reported survey completed by all the participants at baseline and upon completion of the training. Participants were encouraged to complete the survey within 7 days after completion of the intervention. Data at the backend of the system were retrieved to monitor the use of the system (how many times the staff used each chapter and the correctness of the answers to each question).

#### Infection Control Practices Performed by RCH Staff

On-site observation of the infection control practices performed by the RCH staff was the primary outcome of this study. Research nurses were trained to conduct unobtrusive on-site observations in the RCHs when the participants were conducting direct care to the residents (older adults). Unobtrusive means the research nurse would not interfere with the participants’ performance during observation even though the practices were not in line with the infection control guidelines. To minimize bias, each RCH was observed for at least 200 moments at baseline and another 200 moments post intervention. More than one observation session was conducted by the trained research nurse in each RCH in order to obtain at least 200 moments.

An e-rub system was developed by a Swedish company based on the WHO guideline [29], and it was adopted to support the recordings of the observations in the RCHs. The following five infection control practices were observed: (1) hand wash, (2) hand rub, (3) use of gloves, (4) use of PPE, and (5) time spent on hand wash. Haze factors such as wearing rings, bracelets, watches, and long sleeves were also logged in the system. Each observation moment was judged by the research nurse as to whether it was performed properly, improperly, missing, or not applicable. Considering the actual practice in RCHs, “hand hygiene” was defined as “proper hand wash or hand rub.” Proper hand wash referred to hand washing that lasted for 20 seconds or more. Hand rub refers to the use of 70% alcohol for rubbing both hands. We created 2 binary outcome variables based on the on-site observations, namely “use of gloves and PPE and performing respiratory hygiene” and “hand rub,” with 2 options of properly performed versus improperly performed. For the first variable, “properly performed” refers to the situation that the participants had properly used the gloves, performed respiratory hygiene (ie, covered mouth and nose with the upper part of the sleeve while coughing or sneezing) [30], and used the PPE, while for the second variable, “hand rub,” properly performed refers to the situation that the participants did the hand rub properly. The variable, hand rub, is of particular importance, as the staff could not change their gloves and wash hands all the time during the pandemic. Observation moments varied according to the complexity of the care given to the residents; therefore, the total number of moments in each observation varied.

Training was provided to the research nurses to ensure consistent data collection. They were qualified registered nurses with rich experience in patient care and infection control training. All of them were introduced to the e-rub system and were invited to rate the procedures on 4 training videos covering oral feeding, Ryle’s tube feeding, turning handling, and manual handling. Debriefing was given to the research nurses right after the training, ensuring they understood how to rate the observation moments and what we meant by unobtrusive observations. Before they went for on-site assessments, a briefing meeting was held by the project coordinator with the research nurse and to introduce the staff profile of the RCHs. In addition, biweekly regular meetings were conducted with the research nurses and the research team coordinator to ensure the quality of data collected in the RCHs. The research nurses recorded observations at any time in different shifts (morning shift or afternoon shift) on different days (Monday to Saturday), as this arrangement captured a variety of staff behavior at different times. Observations were made at any time without notifying the RCH staff, and they were conducted anywhere in the RCHs.

#### Self-Reported Infection Control Practices

Self-reported infection control practices were measured with the 10-item self-administered questionnaire developed by O’Neil and colleagues [28], asking the participants to indicate whether they and their coworkers often performed the infection control practices, which included hand hygiene before entering and leaving the residents’ rooms, whether they educated the RCH residents about hand and respiratory hygiene, and whether the staff stayed at home if they had respiratory infection symptoms. A 5-point Likert scale with 5 = always, 4 = usually, 3 = sometimes, 2 = rarely, and 1 = never perform was given for selection. A total score was created by summing the scores of all 10 items, with higher scores indicating more frequent performance of the infection control practices. The questionnaire has been used in a large-scale study on health care workers in China [31].

#### Knowledge and Attitudes Toward Infection Control

Knowledge and attitudes toward infection control was measured using the 24-item self-administered instrument developed by O’Neil et al [28], with 12 items about knowledge and 12 items on attitudes in the following four areas: (1) clinical burden and the transmission of acute respiratory infection (7 items), (2) PPE and hand hygiene (6 items), (3) facility infection prevention and control policies for acute respiratory infection (6 items), and (4) influenza vaccination (5 items). Since the last 5 items are not relevant to COVID-19, we did not include these items in the survey. Respondents responded to the 19 statements using a 5-point Likert scale (from strongly disagree to strongly agree). For each knowledge item, respondents were classified as (1) “having knowledge” if their answers were either “strongly agree” or “agree” to the positive statements and “strongly disagree” or “disagree” to the negative statements, or (2) “lack of knowledge” if otherwise. For each attitude item, respondents were classified as (1) “positive attitudes” if their answers were either “strongly agree” or “agree” to the positive statements and “strongly disagree” or “disagree” to the negative statements, or (2) “negative attitudes,” if otherwise. This instrument has also been included in the large-scale study on health care workers in China [31].

#### Total Number of BGCTS Visits

Based on the data retrieved from the backend of the system, we counted the total number of times the health care workers viewed the chapters of the BGCTS.

Demographics of the participants, including age, sex, educational level, profession, previous training in infection control, and months of working experience in the current RCH, and information of the RCH, including financial type, number of staff, and number of residents, were also collected in the baseline survey (Multimedia Appendix 3). Only age and months of working experience were treated as continuous variables in the analysis.

### Data Analyses

To compare sample characteristics at baseline between the intervention and control groups, for categorical variables at the cluster level, Fisher’s exact tests were used, while for variables at the individual level, chi-square tests for categorical variables and independent 2-sided *t* tests for continuous variables were used. Cluster-level analysis was used to examine the between-group difference in the changes in the proportion of infection control practices by on-site observations, with RCHs as clusters. The analysis included a 2-stage approach consisting first of estimating the proportions of the 2 infection control practices (proper use and hand rub) by cluster, and then a weighted cluster-level linear regression was fitted on these proportions. The weights in the regression were set to the number of staff in the RCH and the number of residents in the RCH as a covariate. Demographic variables that have statistical significance between groups at baseline were also included in the regression. The financial type of RCH was not included in the model because of collinearity in the regression model. The distribution of the total number of BGCTS visits among the intervention participants was summarized using descriptive statistics.

To estimate the intervention effect on the self-reported secondary outcomes, we followed Van Breukelen’s [32] recommendation for cluster RCT with pretest and posttest using linear mixed effects models. At the individual level, analyses were performed on the change scores within person (ie, postintervention score minus baseline score) for repeated measures, with a fixed effect for group, a random intercept for cluster, and control for 6 individual-level covariates (sex, age, educational level, baseline staff position, whether they had an infection control program before, and working experience in the current RCH) and 3 cluster-level covariates (number of staff, number of residents, and financial type of RCH) as fixed effects. The intervention effect was reflected by the coefficient of the group. We also conducted sensitivity analysis for self-reported secondary outcomes using multiple imputation to impute missing values using chained equations in regression models that included outcomes for each cluster. This group-based multiple imputation technique performs quite well for the estimates of the fixed effects, which are the primary interest of the study, although it also artificially inflates the true variation between groups [33,34]. A total of 500 imputed datasets were generated. The pooled estimates from the linear mixed-effects model analyses and the corresponding 95% CI were calculated based on the 500 imputed datasets. The analyses were performed using SPSS software (version 30.0; IBM Corp).

### Ethical Considerations

Ethical approval was obtained from the Hong Kong Polytechnic University Institutional Review Board (reference number HSEARS20200903007). Offline informed consent from participants was obtained after a thorough explanation, which was given via Zoom meeting (Zoom Communications, Inc). The intervention participants knew which intervention was the intervention of interest, as they were granted access to the BGCTS, while the control participants also knew they were not given the intervention of interest (BGCTS). Since this intervention lasted for a short period of time (2 weeks only), the control participants did not consider they were in a disadvantage. Privacy consideration was reassured by providing a unique username and password to each participant, and the data were stored in a secured server with no one (except the study team) able to access the data. The nurse manager was given aggregated data of the performance of their staff (individual performance was not disclosed to the nurse managers).

The training system (BGCTS) was demonstrated to the staff via Zoom meetings, as many RCHs closed the door during the COVID-19 pandemic. The study team explained the features of the BGCTS, the planned duration of the training, and the access frequency of the system during the designated period and demonstrated the access method. All this information was included in the written information sheet as well, which was distributed to the staff when they signed the written consent forms.

To protect the privacy of the RCH staff, the staff in the RCH were deidentified and anonymized, and all direct nursing care actions were examined in confidentiality for patients’ conditions. Since the research nurses stayed in the RCH for 2-3 weeks, this design minimized the Hawthorne effect due to observations (ie, RCH staff perform differently when being observed) because the observer’s presence gradually becomes insignificant to the RCH staff [22]. In an RCH unit, more than one health care personnel provided care to the residents. Observations were made on a random basis. There was no interference with the care procedure during the observations. Similar to this previous study [22], no observations would be made if the residents or the relatives of the residents refused to allow the observations around.

## Results

### Overview

**Figure 1:**
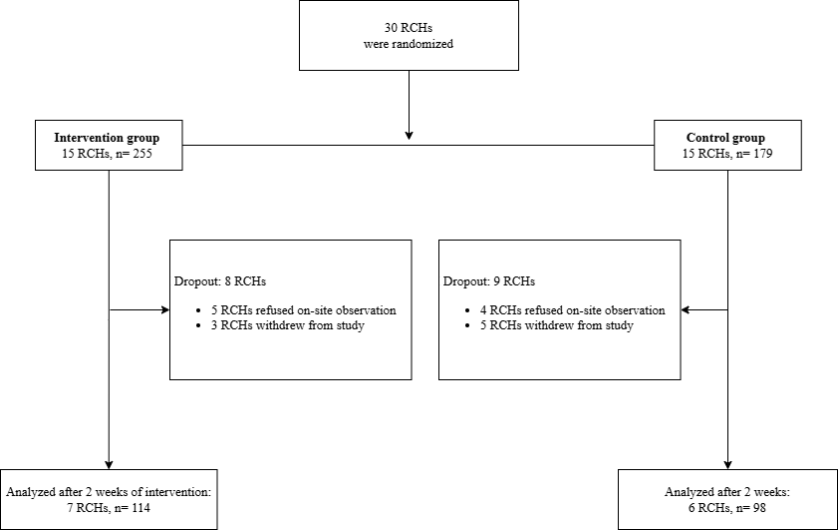
The Consolidated Standards of Reporting Trials diagram of recruitment and data collection. RCH: residential care home.

A total of 30 RCH units were recruited and randomized into either the intervention group (15 RCHs and 255 health care workers) or the control group (15 RCHs and 179 health care workers). Since COVID-19 infection happened during the study period, 9 RCHs did not undergo observations, 2 RCHs delayed the postintervention observations (postintervention observations were conducted 2 months after intervention), 2 RCHs had only 1 observation session with only 20-30 moments in postintervention observations, and 4 RCHs withdrew from the study. Figure 1 shows the CONSORT diagram of recruitment and data collection. A total of 212 participants were recruited from the 13 RCHs, with 114 in the intervention group and 98 in the control group, and included in the analysis. All residents in the 13 RCHs allowed the observations around.

The participating RCHs were similar in terms of number of residents, number of staff, and financial type, with no statistical significance between the 2 groups. At the individual level, the majority of the participants were female, were in their middle age on average, had secondary school education, and were nonprofessional health care workers. Many of them had previous training in infection control and had been working in the current position for years (Table 1). There was no significant difference between the intervention and control groups in all variables at the individual level, except that the proportion of participants having previous training in infection control was significantly larger in the control group (*χ*^2^_1_=13.8; *P*<.001). Post intervention, a total of 142 (67%) participants had completed the survey after training, with 63 (55.3%) in the intervention group and 79 (80.6%) in the control group, and the difference was statistically significant (*χ*^2^_1_=15.3; *P*<.001). The completers were similar to those who were lost to follow-up, except that a significantly larger proportion of completers had previous training in infection control (180/142, 76.1% vs 43/70, 61.4%; *χ*^2^_1_=4.9; *P*=.04). No adverse events were reported during the study period.

**Table 1:** Demographics of the participants in the survey.

Characteristics				Intervention group, n (%)	Control group, n (%)			Statistical test values			*P* value	
**Cluster level (RCH^a^; n=7 intervention group and n=6 control group)**												
	**Number of staff**								—^b^			.82
		<99	4 (57.1)			4 (56.7)						
		100-199	2 (28.6)			2 (33.3)						
		≥200	1 (14.3)			0 (0)						
	**Number of residents**								—^b^			.21
		<50	4 (57.1)			1 (16.7)						
		50-100	0 (0)			2 (33.3)						
		>100	3 (42.9)			3 (50)						
	**Financial type**								—^b^			.59
		Government subsidized	2 (28.6)			3 (50)						
		Private organization	5 (71.4)			3 (50)						
**Individual level (n=114 intervention group and n=98 control group)**												
	**Sex**								0.006 (1)^c^			.94
		Male	12 (10.5)			10 (10.2)						
		Female	102 (89.5)			88 (89.8)						
	**Education**								2.4 (2)^c^			.31
		Secondary school	70 (61.4)			63 (64.3)						
		Postsecondary school	15 (13.2)			8 (8.2)						
		University or above	14 (12.3)			7 (7.1)						
	**Profession^d^**								0.5 (1)^c^			.57
		Nurses or therapists	19 (16.7)			13 (13.3)						
		Nonprofessional workers	95 (83.3)			85 (86.7)						
	**Previous training in infection control**								13.8 (1)^c^			<.001
		Yes	69 (60.5)			82 (83.7)						
		No	45 (39.5)			16 (16.3)						
Age (years), mean (SD)				47.06 (12)	48.89 (11.78)			0.059 (190)^e^			.81	
Working experience in the current RCH (months), mean (SD)				64.20 (61.72)	82.65 (83.13)			–1.754 (162.8)^e^			.08	

^a^RCH: residential care home.

^b^Fisher exact test (statistical values are not applicable).

^c^Chi-square (*df*).

^d^Nonprofessional workers include care-related support workers, health workers, personal care workers, physiotherapy assistants, and occupational therapy assistants who do not have medical-related professional licenses.

^e^*t* test (*df*).

### Infection Control Practices (Primary Outcome)

A total of 5168 moments were observed; 2666 moments (1273 at baseline and 1393 post intervention) were observed in the intervention group, while 2502 moments (1237 at baseline and 1265 post intervention) were recorded in the control group. Proper use of gloves and PPE and performed respiratory hygiene was not common in the RCHs, with 3.1% (37/1197) in the intervention group and 8.3% (97/1168) in the control group at baseline. The proportions of moments showed an increase to 9.3% (124/1339) in the intervention group after the BGCTS training, while a decrease to 5.4% (67/1246) in the control group after the usual care training. On the other hand, the proportion of moments with hand rub performed properly at baseline was high in both groups, with 89.5% (1139/1273) in the intervention group and 96% (1187/1237) in the control group. After training, the proportion increased to 94.9% (1322/1393) in the intervention group, while it remained similar in the control group (Table 2). In addition to the number of residents in the RCH, the proportion of participants having previous training in infection control in the RCH was also included in the weighted cluster-level regression. Results of the weighted cluster-level regressions also supported that the BGCTS had a beneficial effect on both objective primary outcomes when compared to the usual care of the infection control program (Table 2). The BGCTS group had increased significantly more in the proportion of proper use of gloves and PPE and performed respiratory hygiene than the control group (β=.195, 95% CI 0.046-0.344; *t*_1_=2.962; *P*=.02) and significantly more in the proportion of hand rub in the intervention group than that in the control group (β=.068, 95% CI 0.005-0.132; *t*_1_=2.445; *P=*.04). Among the intervention participants, the mean of the total number of BGCTS visits was 7.68 (SD 6.92; median 8, range 0-52). A total of 23 (20.18%) participants did not visit any of the 8 BGCTS chapters, and 18 (78.26%) of them had previous training in infection control.

**Table 2:** Results from analyses of the primary outcomes, use of gloves and personal protective equipment (PPE) and perform respiratory hygiene and hand rub between the intervention and control groups.

Outcomes		Intervention group, n (%)			Control group, n (%)			Weighted cluster-level linear regression^a^					
		Baseline	Post intervention	Baseline		Post intervention	β (95% CI)			*t* test (*df*)		*P* value	
**Use of gloves and PPE and perform respiratory hygiene**									.195 (0.046-0.344)		2.962 (1)		.02
	Improperly performed	1160 (96.9)	1215 (90.7)	1071 (91.7)		1179 (94.6)							
	Properly performed	37 (3.1)	124 (9.3)	97 (8.3)		67 (5.4)							
**Hand rub**									.068 (0.005-0.132)		2.445 (1)		.04
	Improperly performed	134 (10.5)	71 (5.1)	50 (4)		36 (2.8)							
	Properly performed	1139 (89.5)	1322 (94.9)	1187 (96)		1229 (97.2)							

^a^The number of residents in the residential care home and the proportion of participants having previous training in infection control in the residential care home were included as covariates.

### Self-Reported Infection Control Practices and Knowledge and Attitudes Toward Infection Control (Secondary Outcome)

Results on comparing the changes in the secondary outcomes in the 2 groups are provided in Tables 3 and 4, on their mean scores at baseline and post intervention and mixed-effects model results, respectively. Table 3 showed that the mean score in self-reported infection control practices increased in the intervention group from 40.23 (SD 5.82) to 42.81 (SD 4.22) while reduced from 42.21 (SD 4.33) to 41.66 (SD 3.88) in the control group after training. Slight increments in mean scores in the 3 outcomes in knowledge and attitudes toward infection control were observed in both groups after training. However, the mixed effects model results revealed that there were no statistically significant differences in the mean score changes between the 2 groups in all 4 secondary outcomes in both the complete data and multiple imputation analyses (Table 4). For other controlling factors, only number of staff was statistically and significantly associated with knowledge about clinical burden and the transmission of acute respiratory infection in the multiple imputation analysis (Table 4).

**Table 3:** Mean and SD of self-reported infection control practices and knowledge and attitudes toward infection control by group and time.

Outcomes	Intervention group, mean (SD)			Control group, mean (SD)	
	Baseline	Post intervention	Baseline		Post intervention
Self-reported infection control practices	40.23 (5.82)	42.81 (4.22)	42.21 (4.33)		41.66 (3.88)
Knowledge about clinical burden and the transmission of acute respiratory infection	27.90 (4.74)	29.05 (4.42)	28.71 (4.56)		29.63 (4.15)
Knowledge about personal protective equipment and hand hygiene	23.17 (4.16)	24.98 (3.48)	24.62 (2.97)		25.49 (2.81)
Knowledge about facility infection prevention and control policies for acute respiratory infection	23.26 (3.45)	23.94 (2.98)	23.83 (3.33)		24.52 (3.43)

**Table 4:** Results of linear mixed effects models on change scores in secondary outcomes (self-reported infection control practices and knowledge and attitudes toward infection control).

Outcomes			Complete data							Multiple imputation				
			β (95% CI)		*t* test (*df*)		*P* value		β (95% CI)			*t* test (*df*)		*P* value
**Self-reported infection control practices^a^**														
	Group	.13 (–8.44 to 8.71)		0.043 (1)		.97		–.92 (–3.63 to 1.80)			–0.663 (1)		.51	
	Sex	–.65 (–3.87 to 2.57)		–0.403 (1)		.69		–1.66 (–4.67 to 1.35)			–01.080 (1)		.28	
	Education	–.61 (–2.28 to 1.06)		–0.729 (1)		.47		–.49 (–1.98 to 0.99)			–0.651 (1)		.52	
	Age	.04 (–0.05 to 0.14)		0.904 (1)		.37		.04 (–0.04 to 0.12)			0.916 (1)		.36	
	Working experience	.00 (–0.01 to 0.02)		0.683 (1)		.50		.00 (–0.01 to 0.02)			0.663 (1)		.51	
	Profession	1.87 (–1.00 to 4.74)		1.292 (1)		.20		1.86 (–1.09 to 4.82)			1.235 (1)		.22	
	Previous infection control training	.38 (–2.16 to 2.92)		0.299 (1)		.77		.49 (–1.91 to 2.88)			0.399 (1)		.69	
	Number of staff	.04 (–0.03 to 0.11)		1.141 (1)		.26		.03 (–0.03 to 0.08)			1.053 (1)		.29	
	Number of residents	.01 (–0.05 to 0.08)		0.502 (1)		.63		.00 (–0.03 to 0.04)			0.138 (1)		.89	
	Financial type	1.51 (–1.07 to 4.09)		1.234 (1)		.23		1.06 (–0.49 to 2.62)			1.338 (1)		.18	
**Knowledge about clinical burden and the transmission of acute respiratory infection^a^**														
	Group	.03 (–4.42 to 4.49)		0.021 (1)		.98		.23 (–1.83 to 2.28)			0.216 (1)		.73	
	Sex	–.30 (–3.28 to 2.68)		–0.201 (1)		.84		–.88 (–3.47 to 1.71)			–0.665 (1)		.51	
	Education	–.30 (–1.85 to 1.25)		–0.380 (1)		.71		–.65 (–1.95 to 0.64)			–0.989 (1)		.32	
	Age	.01 (–0.07 to 0.10)		0.329 (1)		.74		–.01 (–0.09 to 0.07)			–0.235 (1)		.81	
	Working experience	.00 (–0.01 to 0.01)		0.302 (1)		.76		.00 (–0.01 to 0.01)			0.110 (1)		.91	
	Profession	1.05 (–1.62 to 3.71)		0.780 (1)		.44		1.78 (–0.78 to 4.33)			1.363 (1)		.17	
	Previous infection control training	–.04 (–2.35 to 2.27)		–0.033 (1)		.97		.61 (–1.49 to 2.72)			0.573 (1)		.57	
	Number of staff	.05 (–0.01 to 0.10)		1.839 (1)		.10		.05 (0.01 to 0.09)			2.287 (1)		.02	
	Number of residents	–.01 (–0.05 to 0.03)		–0.712 (1)		.50		–.01 (–0.05 to 0.01)			–1.020 (1)		.31	
	Financial type	.55 (–1.37 to 2.47)		0.715 (1)		.50		.83 (–0.33 to 2.00)			1.399 (1)		.16	
**Knowledge about personal protective equipment and hand hygiene^a^**														
	Group	.25 (–4.73 to 5.23)		0.197 (1)		.88		.32 (–2.36 to 2.98)			0.232 (1)		.82	
	Sex	.10 (–2.31 to 2.51)		0.086 (1)		.93		–1.10 (–3.13 to 0.92)			–1.067 (1)		.29	
	Education	–1.20 (–2.45 to 0.06)		–1.894 (1)		.06		–.94 (–1.98 to 0.09)			–1.783 (1)		.08	
	Age	–.04 (–0.11 to 0.04)		–1.009 (1)		.32		–.02 (–0.08 to 0.04)			–0.565 (1)		.57	
	Working experience	.00 (–0.01 to 0.01)		.502 (1)		.62		.00 (–0.01 to 0.01)			0.492 (1)		.82	
	Profession	1.05 (–1.10 to 3.20)		0.968 (1)		.34		.85 (–1.13 to 2.82)			0.840 (1)		.40	
	Previous infection control training	1.21 (–0.67 to 3.09)		1.279 (1)		.20		1.11 (–0.62 to 2.85)			1.257 (1)		.21	
	Number of staff	.03 (–0.02 to 0.08)		1.493 (1)		.18		.03 (–0.01 to 0.07)			1.515 (1)		.13	
	Number of residents	–.01 (–0.50 to 0.02)		–1.052 (1)		.36		–.01 (–0.04 to 0.02)			–0.694 (1)		.49	
	Financial type	–.70 (–2.51 to 1.12)		–1.083 (1)		.34		–.78 (–2.09 to 0.52)			–1.175 (1)		.24	
**Knowledge about facility infection prevention and control policies for acute respiratory infection^a^**														
	Group	.84 (–3.53 to 5.20)		0.550 (1)		.61		.08 (–1.86 to 2.02)			0.079 (1)		.94	
	Sex	–1.67 (–3.64 to 0.31)		–1.677 (1)		.10		–1.43 (–3.51 to 0.65)			–1.346 (1)		.18	
	Education	–.52 (–1.54 to 0.51)		–1.001 (1)		.32		–.23 (–1.33 to 0.87)			–0.408 (1)		.88	
	Age	.01 (–0.05 to 0.07)		0.316 (1)		.75		.01 (–0.06 to 0.08)			0.194 (1)		.85	
	Working experience	.00 (–0.01 to 0.01)		0.539 (1)		.59		.00 (–0.01 to 0.01)			0.287 (1)		.77	
	Profession	–.14 (–1.90 to 1.62)		–0.160 (1)		.87		–.19 (–2.26 to 1.89)			–0.177 (1)		.86	
	Previous infection control training	–.73 (–2.29 to 0.82)		–0.937 (1)		.35		–.83 (–2.56 to 0.90)			–0.937 (1)		.35	
	Number of staff	.03 (–0.01 to 0.07)		1.524 (1)		.14		.02 (–0.02 to 0.06)			1.151 (1)		.25	
	Number of residents	–.00 (–0.04 to 0.03)		–0.308 (1)		.77		–.01 (–0.04 to 0.01)			0.913 (1)		.36	
	Financial type	–.24 (–1.71 to 1.23)		–0.356 (1)		.73		.22 (–0.82 to 1.27)			0.287 (1)		.77	

^a^Six individual-level covariates (sex, age, educational level, baseline staff position, whether had infection control program before, and working experience in the current RCH) and 3 cluster-level covariates (number of staff, number of residents, and financial type of RCH) were included as fixed effects in the linear mixed-effects model.

## Discussion

### Overview

On the basis of the cluster-level analysis, the trial showed benefits of the 2-week blended infection control training BGCTS over usual care of an infection control training session for infection control practices of hand rub and the use of gloves and PPE and proper performance in respiratory hygiene among health care workers in RCHs. The study findings have extended the literature on the effectiveness of educational interventions on infection control practices by providing evidence from more than 5500 on-site observations, rather than data from self-reported questionnaires, as well as evidence in long-term care facilities with an experimental design during an outbreak setting [8,9]. After attending the BGCTS training, more RCH staff in the intervention group performed properly in the use of gloves and PPE and respiratory hygiene and rubbed their hands properly than those in the control group. These infection control practices are crucial to protect the staff from being infected with COVID-19. One of the stories in the BGCTS allowed the staff to choose appropriate PPE for different scenarios in RCHs (please refer to Multimedia Appendix 2 for the key learning points in the BGCTS). This is helpful to the staff and makes them understand the relevant guidelines in glove and PPE use. Wearing insufficient PPE cannot protect the staff, while wearing inappropriate PPE may waste the resources in the RCHs, leading to insufficient resources when they are needed. Educating the RCH staff on how to choose the most appropriate PPE and gloves for particular procedures is crucial in infection control training and making good use of the resources in the RCH facilities [35].

These study findings have provided preliminary evidence that gamification was a good strategy for the infection control practices among the staff in RCHs. This observation echoes the potential benefits of blended learning and active learning in delivering evidence-based health care educational interventions recommended by a recent SR including 61 RCTs [36] and is consistent with the findings from a previous study in which the correct concepts and information regarding the choice and handling of PPE can be delivered to the staff (game players) in interactive manners [37]. In this study, the majority of the participants were nonprofessional staff in the RCHs whose medical knowledge was expected to be lower than that of professional staff. This result implies the wide applicability of the BGCTS in public health training, in particular, to nonprofessional staff who are always the key frontline staff in long-term care settings. The key feature in the BGCTS of the voice-over function in the mother tongue language (Cantonese) in the games has ensured the staff access the information without language barrier or reading demand, which is critical to nonprofessional staff in strengthening their ability to apply knowledge and analytical skills in infection control practices.

Hand hygiene is the key in infection control practices. According to the WHO’s recommendation [29], proper hand wash or hand rub is considered proper hand hygiene. Since RCHs are busy clinical sites in which staff always struggle with time when they provide direct care to the residents, many staff perform hand rubs rather than hand washes. Training about the conditions when hand rub could replace hand wash would be needed, especially in the SARD or COVID-19 condition, where hand wash is nearly impossible when the staff is fully gowned. The percentage of hand rubs at baseline was very high in the control group (1187/1237, 96%), which has limited its ability to demonstrate significant gains in the control participants and potentially underestimated the impact of usual care and thereby overestimated the impact of BGCTS. We noted the percentage of hand rub at baseline in both groups was very high. Such a high hand rub percentage may be due to the occurrence of the COVID-19 pandemic in the study period [38] because the percentage was reported as low as 15% in an observational study in the RCHs in Hong Kong in 2019 [2]. Upon the outbreak of the COVID-19 pandemic, many RCH staff had been educated to pay attention to hand hygiene before we started the BGCTS intervention. Nevertheless, the results suggested that the BGCTS training has good efficacy to promote the staff’s performance in proper hand hygiene, in particular, hand rub.

Although the measures of these 2 outcomes were made objectively, our results might have been confounded by the implementation of the study because the halt or delay of all research activities in some of the participating RCHs due to the COVID-19 pandemic in the study period finally led to the dropout, delay in observations, or insufficient observations in 17 RCHs. The large reduction in the number of clusters from 30 to 13 RCHs has resulted in an underpowered analysis.

In the survey, the intervention group had reported an increase while the control group reported a decrease in the scores of self-reported infection control practices, whereas both groups showed increases in the scores in the 3 knowledge and attitude toward infection control scales, although the between-group differences were not statistically significant. These findings were in line with the literature that self-reported knowledge, attitude, and practices increased after receiving educational training [10-13]. The nonsignificant results could be attributed to several possible reasons in the design and the implementation of the study. First, the use of active control may have produced improvements in knowledge, attitude, and practices in infection control in the control participants. During the COVID-19 pandemic, staff in RCHs were at high risk of infection, and hence they would treat these briefing sessions more seriously and pay more attention to infection control, which may result in self-searching of infection control knowledge. Second, the significant between-group attrition rates post intervention might have introduced attrition biases. Third, the reduced sample size had lowered the study power in detecting between-group differences. Another possible reason was the measurement tools themselves. The measurement tools for the self-reported infection control practices and the 3 knowledge and attitude toward infection control scales have been used in infection control practice studies [31]; however, their psychometric properties have not yet been reported [28], and the use of self-reported questionnaires for outcome assessment also may lead to response bias by providing socially desirable responses from the participants. Thus, these findings might be subject to measurement error. We recommend that the psychometric properties of these scales be fully examined before their use in future studies. Meanwhile, these nonsignificant findings also probably reflected that the contents of the BGCTS training have not well addressed all the issues mentioned in these scales; for example, the clinical burden of acute respiratory infection and the policies in relation to facility infection prevention and control. Since infection control practices cover a broad spectrum of knowledge, showing a broad array of pathogens and different modes of transmission across various settings, the intensity of gamification in the BGCTS may not be sufficient to cover all these. Alternatively, these nonsignificant results might also be attributed to the low engagement in the BGCTS in some of the intervention participants. Increasing the contents or developing more videos and games could be considered to foster learning. Further studies are warranted to test this claim.

### Limitations

This study has some limitations. The main study limitation was that the trial was carried out when Hong Kong experienced the fifth wave of the COVID-19 pandemic, and the recruitment was severely affected, leading to less satisfactory power in the analysis. Many RCHs refused the on-site observations or turned down the postintervention observations due to infected cases in the homes. This did not only induce attrition bias but also reduced the sample size for analysis, leading to the underpower of the study as well as the reduced generalizability of the study findings. Second, the occurrence of the pandemic may have altered the performance of the RCH staff in infection control, leading to a ceiling effect of hand rub. The findings about hand rub should further be investigated in postpandemic or nonpandemic periods when the RCH staff become less stressed so that their usual practices can be captured in observations. Third, the attrition bias might be further compounded by the significantly higher attrition rate post intervention in the intervention group as well as the fact that significantly more completers had previous training in infection control, leading to an unpredictable direction of the attrition bias. Fourth, the study findings on the 4 secondary outcomes might also be subject to measurement errors because they were assessed by the use of self-reported questionnaires, including measurement tools with unclear psychometric properties. Lastly, there may be observer bias, Hawthorne effects, or interrater reliability among research nurses in collecting objective on-site measurements, although we have attempted to minimize these biases by providing training in data collection to the nurses, taking the observations on a random basis, and making sure there is no interference with the care procedure during the observations.

### Recommendations

This study has implications for practices and future research. First, this is the first-of-its-kind electronic platform for infection control training. With the current evidence from this study, the system should be further tested with studies to be conducted in the postpandemic condition. Nevertheless, this BGCTS training still can be considered as a tool for either training for new staff or reinforcement training for existing RCH staff. Since this system provides data about staff’s engagement in the infection control training, the management team of the RCHs can use these data to plan for reinforcement training workshops for the staff. For future research, this study is a good foundation for understanding the efficacy of e-learning in RCH staff development. The study design of this study is robust with the collection of objective and subjective data and thus can be used as a reference for future research in other clinical settings.

Implementing good infection control measures and practices in RCHs protects residents and staff in the RCH from being infected. It was very unfortunate that we had a very high mortality rate for older people in the fifth wave of COVID-19 in early 2022, with 87% of the cumulative deaths being people aged 70 years or above [4,39]. RCHs were the most traumatized places, where many residents were infected and deceased due to COVID-19. This tragedy demonstrates the weakness of traditional infection control training in RCHs, in which lectures or educational talks are usually given to the staff. The assumption has been made that the RCH staff understand the infection control guidelines after the talks and carry out the infection control measures as told. It is uncommon to check their interpretation and keep track of their practices after training. This study provides some preliminary evidence that the BGCTS is a potentially good model of infection control training that could be applied in the RCHs. The BGCTS provides a record of the training, and this could identify who needs more training. Building up the RCH staff’s competence in infection control practice prevents the spread of infectious diseases among the residents and infectious outbreaks in the RCHs.

### Conclusions

In conclusion, the BGCTS training was shown to better improve actual performance in infection control practices regarding “the use of gloves and PPE and respiratory hygiene” and “hand rub” when compared to the usual care training, usually in terms of lectures or poster self-learning. However, the BGCTS did not perform better than infection control briefing sessions in self-reported infection control practices as well as knowledge and attitude toward infection control in health care workers in RCHs.

## Appendix

Multimedia Appendix 1 CONSORT-eHEALTH checklist (V 1.6.1).

Multimedia Appendix 2 The 8 stories in the Blended Gaming COVID-19 Training System.

Multimedia Appendix 3 The demographic questionnaire used in the study.

## Abbreviations

BGCTS: Blended Gaming COVID-19 Training System
CONSORT: Consolidated Standards of Reporting Trials
PPE: personal protective equipment
RCH: residential care home
RCT: randomized controlled trial
SARD: severe acute respiratory diseases
SR: systematic review
WHO: World Health Organization

## Data Availability

The datasets generated and analyzed during this study are available from the corresponding author on reasonable request. The Blended Gaming COVID-19 Training System is now available to be subscribed to by residential care homes or nursing schools according to the number of users in the institutions. The fee covers unlimited access to the system within the period, 2 administrator accounts for monitoring the staff and students’ use, and the summary note of the performances of the users. The fee will be waived for collaborative research.
