# Supplementary material for: Efficacy of the Web-Based Gamified Infection Control Training System on Practices for Health Care Workers in Residential Care Homes: Clustered Randomized Controlled Trial
Source: JMIR Serious Games. 2025 Nov 27;13:e71593. doi: 10.2196/71593 (PMC12699250; doi:10.2196/71593)
Supplement: Multimedia Appendix 2 [file games_v13i1e71593_app2.docx]

| **Stories of the BGCTS system** | **Guidelines from WHO and local health authority** | | **Example of the print screen** | | **Translations** |
| --- | --- | --- | --- | --- | --- |
| Story 1. Preparing for COVID-19 at your healthcare facility.  Highlighted areas:  Triage procedures, social distancing and suggested policy for visiting hours. | **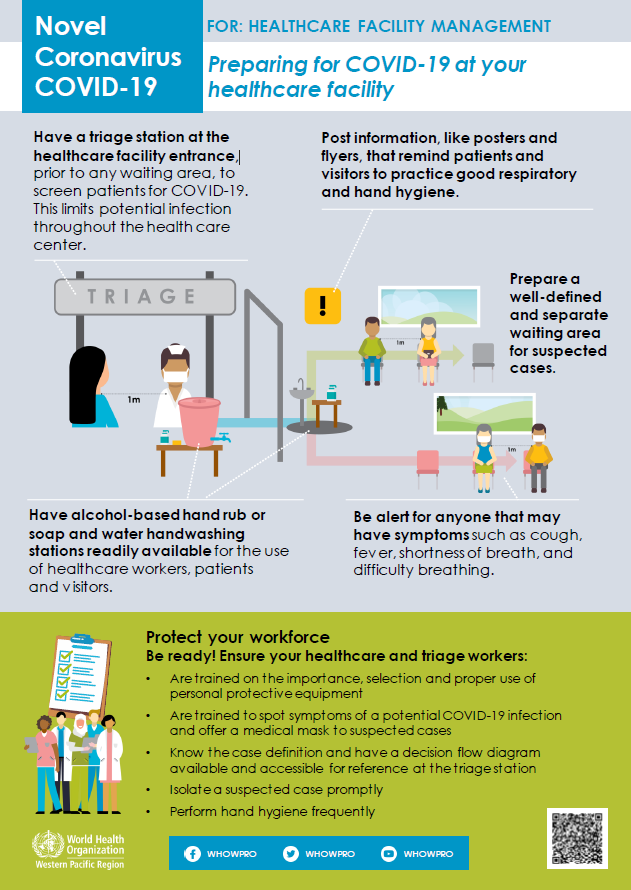** | | **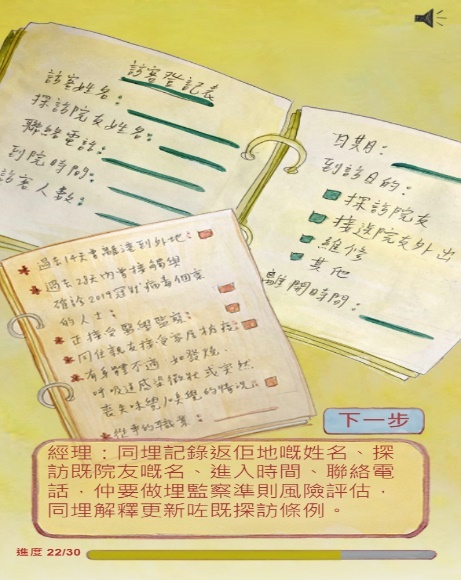** | | Booklet (top to bottom)   1. Visitor registration form  - Name - Visiting patient - Phone number - Visiting time - Number of visitors - Date - Purpose of visit - Visit - Transportation - Maintenance - Others - Departure time  1. Risk assessment  - Have you been to foreign countries in the past 14 days - Any contact with people who contracted COVID 19 in the past 28 days - Are you currently under medical observation - Any cohabiting friends or relatives who are under quarantine. - Any bodily discomfort, such as fever, respiratory infection symptoms, or loss of taste/ smell - Occupation:___   Manager:  Remember to log down their (visitors’) names, who they are visiting, the time of visit, and contact information. You would also need to complete the epidemiology risk assessment and explain the most up-to-date visitation policy. |
| Story 2. Managing patients with suspected or confirmed COVID 19 at your healthcare facility  Highlighted areas: Handling of suspected or confirmed cases of COVID-19 and environmental cleaning | **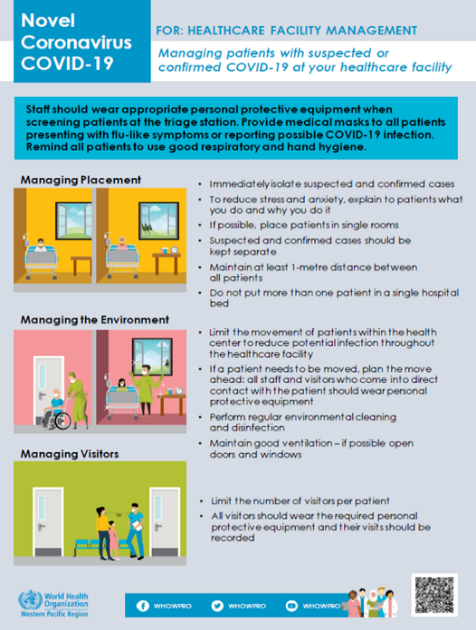** | | **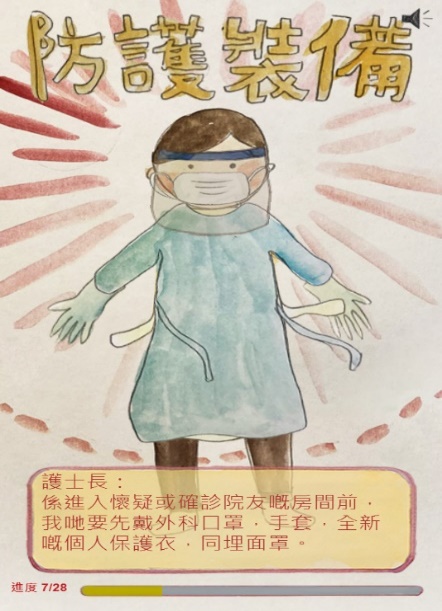** | | Title: Protective equipment  Nursing Officer:  Make sure to wear your surgical mask, gloves, new personal protective equipment, and a face shield before you enter a suspected or confirmed COVID 19 patient’s room. |
| Story 3. Protecting yourself at work from COVID-19  Highlighted area:  Proper use of PPE | **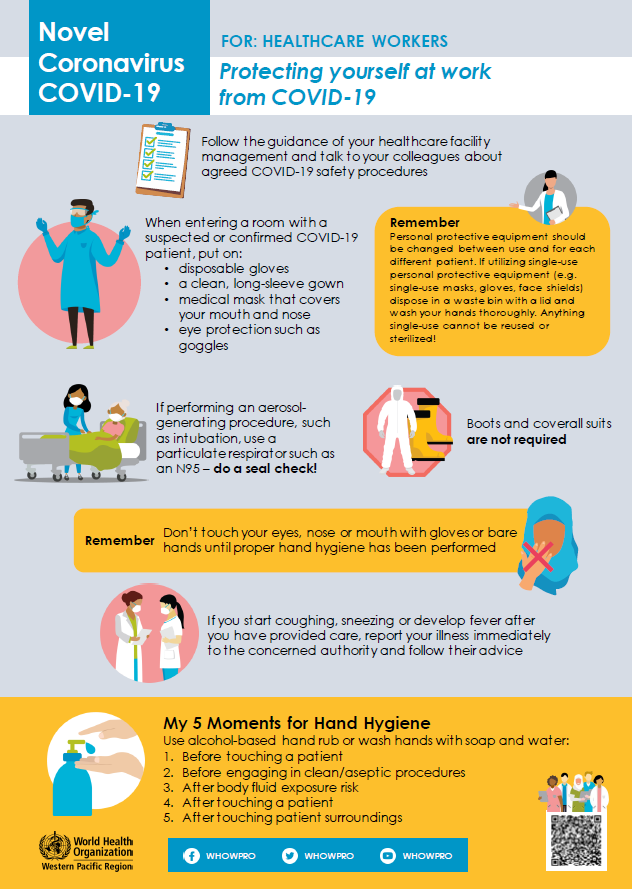** | | **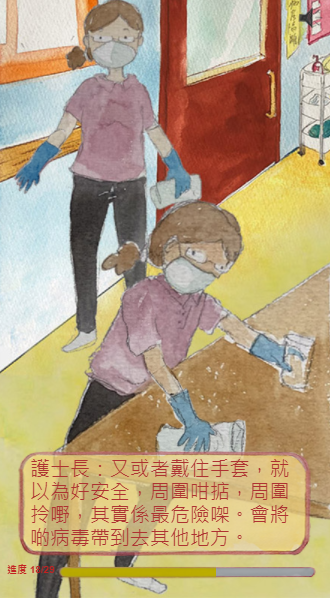** | | Nursing officer: Or you’re wearing gloves, thinking it’s safe to touch or hold things in your surroundings. It’s actually the most dangerous, since you would carry the virus into other places. |
| **Stories of the BGCTS system** | | **Guidelines from WHO and local health authority** | | **Example of the print screen** |  |
| Story 4 Personal Protective Equipment (PPE) According to Healthcare Activities | | **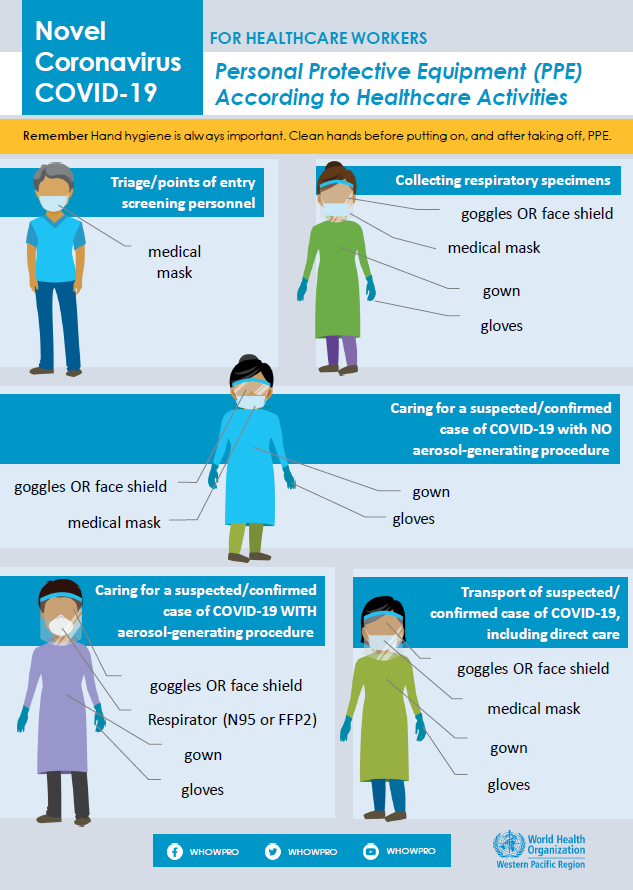** | | **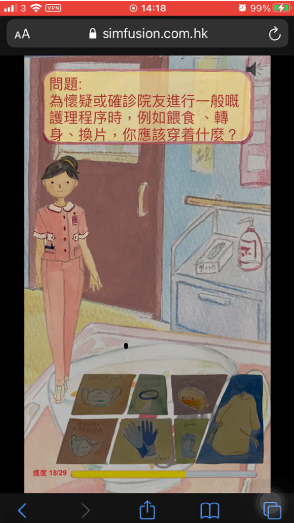** | Question: What should you wear when you’re performing routine bedside care, such as feeding, turning, or changing diapers, for a suspected/ confirmed patient of COVID 19? |
| Story 5. Communicating with patients with suspected or confirmed COVID-19.  To introduce appropriate communication techniques when communicating with patients with suspected or confirmed COVID 19 | | **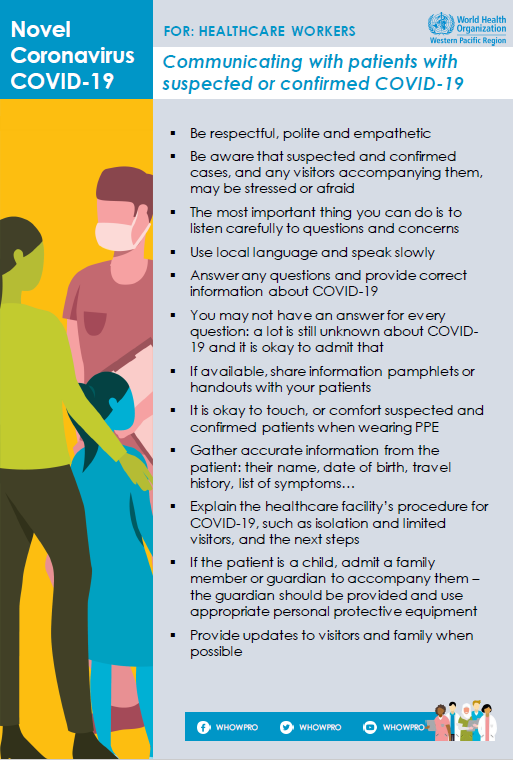** | | **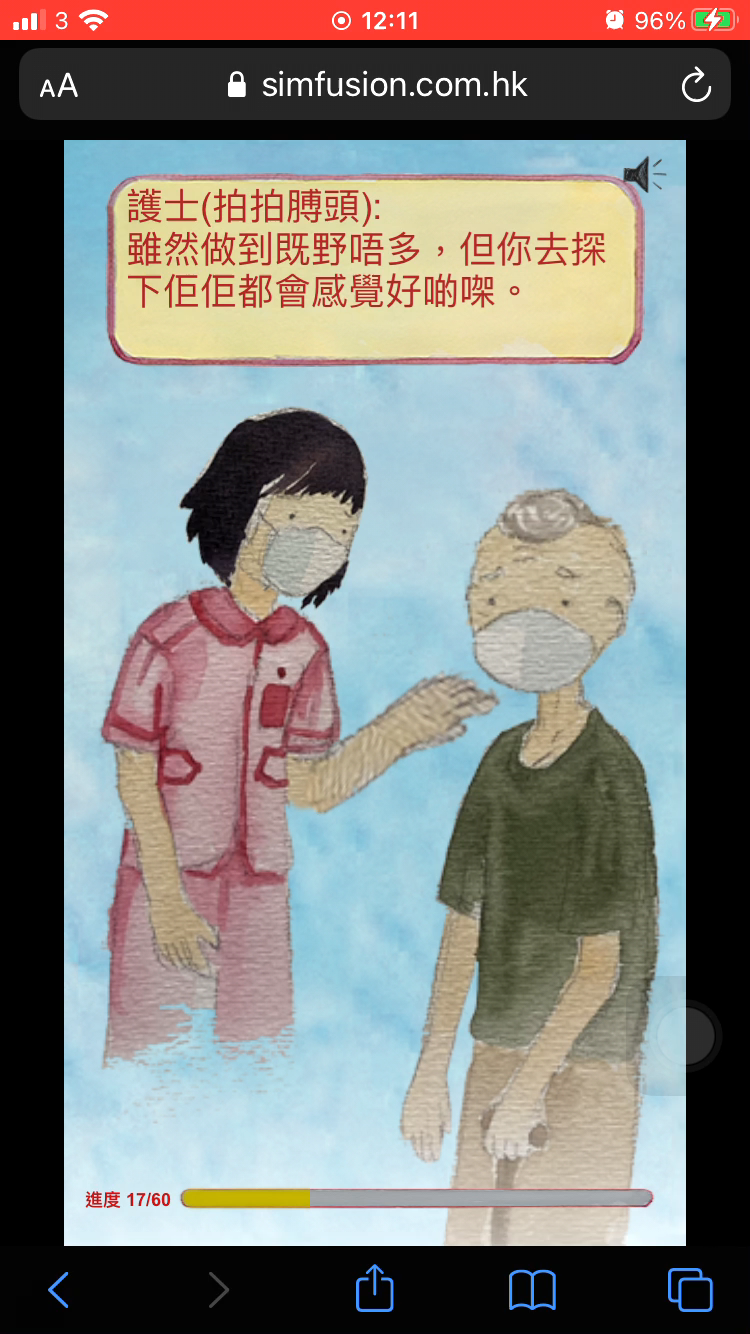** | Nurse (petting his shoulder):  Although there’s not much you can do, they’d feel better if you visit them. |
| Story 6. Information about COVID-19   - Sign and symptoms Environmental cleaning - Preventive measures - Means of transmission - Hand and respiratory hygiene | | **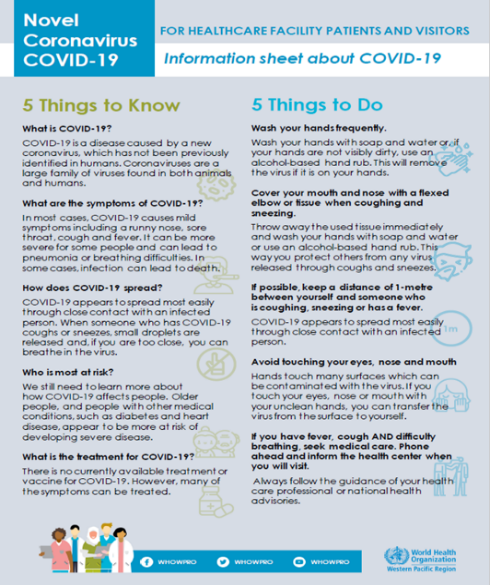** | | **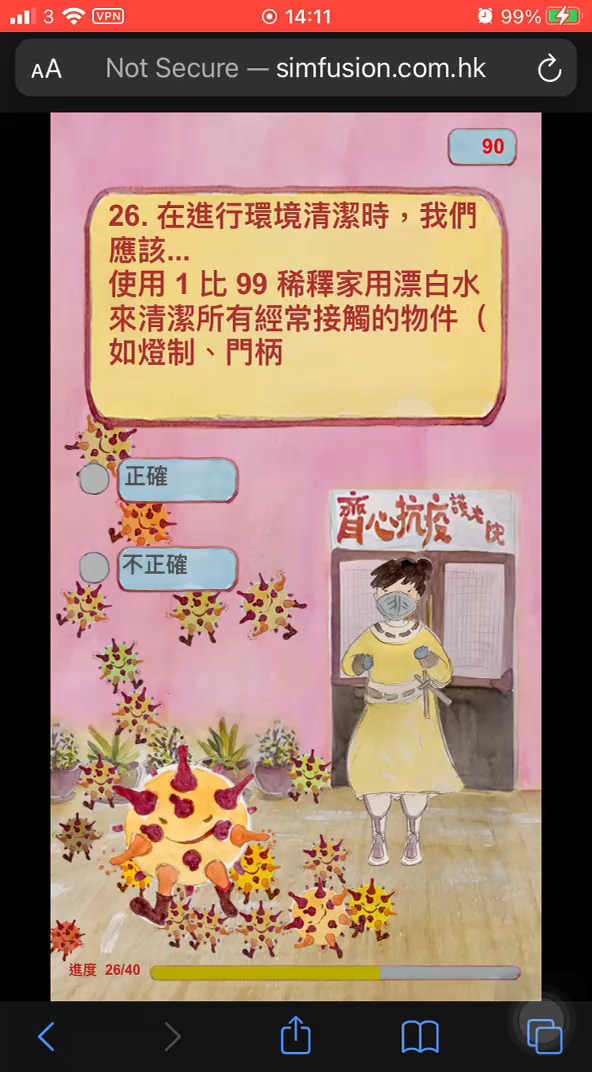** | 26. When performing environmental cleaning, we should use 1:99 diluted bleach to clean frequently touched objects, such as light switches and door handles.   - Correct - Incorrect |
| Story 7. Coping with stress   - To introduce appropriate ways of stress coping | | **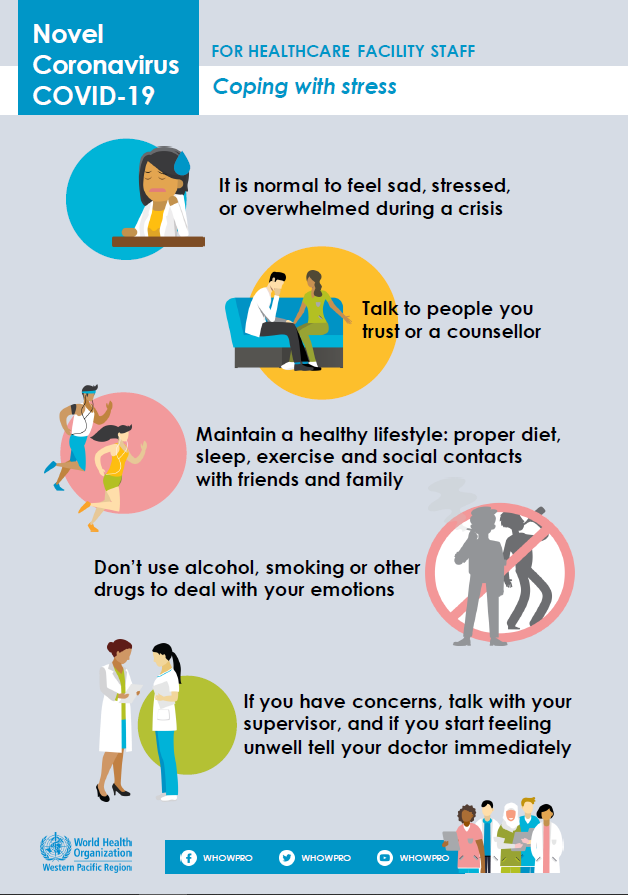** | | 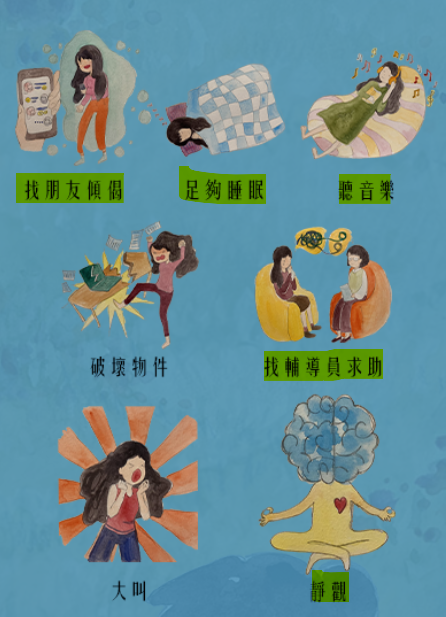 | (From left to right and top to bottom:)   1. Chatting with friends 2. Sufficient sleep 3. Listen to music 4. Breaking things 5. Seek help from counsellors 6. Yelling 7. mindfulness |
| Story 8. My 5 moments for hand hygiene   - To introduce different Hand Hygiene opportunities and their techniques with video appraisal & games | | 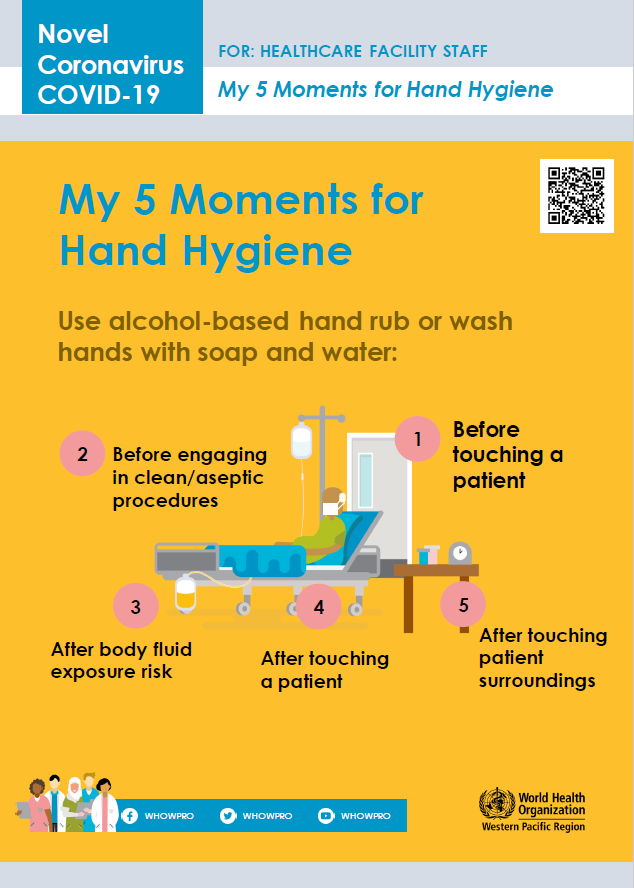 | | **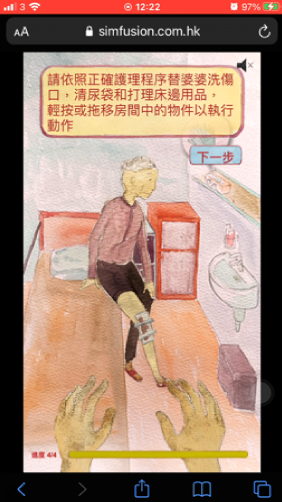** | Please follow the proper way of wound cleaning, empty the urinary bag, and tidy up the bedside cupboard. Press or drag appropriate objects to proceed |
