# Supplementary material for: Efficacy of the Web-Based Gamified Infection Control Training System on Practices for Health Care Workers in Residential Care Homes: Clustered Randomized Controlled Trial
Source: JMIR Serious Games. 2025 Nov 27;13:e71593. doi: 10.2196/71593 (PMC12699250; doi:10.2196/71593)
Supplement: Multimedia Appendix 3 [file games_v13i1e71593_app3.pdf]

Demographics questions

1. Age: ____________

Please check the appropriate box(es) that applies

2. Gender
    - o Male
    - o Female

3. Educational level
    - o Primary school
    - o Secondary school
    - o Post-secondary college
    - o Undergraduate degree
    - o Master’s degree
    - o PhD
    - o Others:___

4. Occupation
    - o Physician
    - o Registered nurse
    - o Registered nurse (Psychiatric)
    - o Enrolled nurse
    - o Physiotherapist
    - o Occupational therapist
    - o Care-related support worker
    - o Health worker
    - o Personal care worker
    - o Care worker
    - o Physiotherapy assistant
    - o Occupational therapy assistant
    - o Others:___

5. Type of residential care home you work in
   - Care and attention place
   - Nursing home
   - Home for the aged
   - Residential respite service
   - Others:___

6. Funding source for your current residential care home
   - Social welfare department
   - Other governmental departments
   - NGOs
   - Self-financed
   - Private companies
   - Not sure
   - Others:___

7. How long have you been working here?

   Years:___ Months:___

8. Current staff-to-resident ratio

   Number of staff:___ Number of residents:___

9. How long have you been working in other care homes (if applicable)?

   Years:___ Months:___

10. Have you attended any infection control training before?
    - Yes
    - Never

Please answer the follow questions based on your most recent training content.

11. Training duration (hours):_____
12. Date(dd/mm/yy):___
13. Training content (multiple choice)
    - ○ Hand Hygiene knowledge and operations
    - ○ Donning of protective personal equipment
    - ○ The application and types of masks
    - ○ N95 fit tests
    - ○ Epidemiology risk assessment (FTOCC)
    - ○ Handling of medical waste and sharps disposal
    - ○ Environment cleaning and disinfection
    - ○ Arrangement and policy for resident with contact/ droplet/airborne precaution
    - ○ Others: (please specify) ______________________________________
